# LncRNA MEG3: Potential stock for precision treatment of cardiovascular diseases

**DOI:** 10.3389/fphar.2022.1045501

**Published:** 2022-11-29

**Authors:** Zining Li, Jialiang Gao, Di Sun, Qian Jiao, Jing Ma, Weilu Cui, Yuqing Lou, Fan Xu, Shanshan Li, Haixia Li

**Affiliations:** ^1^ Guang’anmen Hospital, China Academy of Chinese Medical Sciences, Beijing, China; ^2^ Master’s Degree Student, Beijing, China; ^3^ Cardiovascular Division, Beijing, China; ^4^ Deputy Chief Physician, Beijing, China; ^5^ Chief Physician, Beijing, China

**Keywords:** lncRNA MEG3, cardiovascular diseases, P53 signaling pathway, PI3K/akt signaling pathway, apoptosis, autophagy, inflammation, endoplasmic reticulum

## Abstract

The prevalence and mortality rates of cardiovascular diseases are increasing, and new treatment strategies are urgently needed. From the perspective of basic pathogenesis, the occurrence and development of cardiovascular diseases are related to inflammation, apoptosis, fibrosis and autophagy of cardiomyocytes, endothelial cells and other related cells. The involvement of maternally expressed gene 3 (MEG3) in human disease processes has been increasingly reported. P53 and PI3K/Akt are important pathways by which MEG3 participates in regulating cell apoptosis. MEG3 directly or competitively binds with miRNA to participate in apoptosis, inflammation, oxidative stress, endoplasmic reticulum stress, EMT and other processes. LncRNA MEG3 is mainly involved in malignant tumors, metabolic diseases, immune system diseases, cardiovascular and cerebrovascular diseases, etc., LncRNA MEG3 has a variety of pathological effects in cardiomyocytes, fibroblasts and endothelial cells and has great clinical application potential in the prevention and treatment of AS, MIRI, hypertension and HF. This paper will review the research progress of MEG3 in the aspects of mechanism of action, other systemic diseases and cardiovascular diseases, and point out its great potential in the prevention and treatment of cardiovascular diseases. lncRNAs also play a role in endothelial cells. In addition, lncRNA MEG3 has shown biomarker value, prognostic value and therapeutic response measurement in tumor diseases. We boldly speculate that MEG3 will play a role in the emerging discipline of tumor heart disease.

## 1 Introduction

The prevalence and mortality rates of cardiovascular diseases are on the rise, with an estimated 19 million people worldwide dying from cardiovascular diseases in 2020, an increase of 18.7% from 2010 (Tsao et al., 2022). 80% of deaths are related to CVD (Global, 2017). By 2030, 23.6 million people are expected to die from cardiovascular disease, including heart disease and stroke (Dehghan et al., 2017), (Yusuf et al., 2014). New treatment strategies are urgently needed. From the perspective of basic pathogenesis, the occurrence and development of cardiovascular diseases are related to inflammation, apoptosis, fibrosis and autophagy of cardiomyocytes, endothelial cells and other related cells (Hou et al., 2014; Zhang et al., 2019a; Lan et al., 2019; Shihabudeen Haider Ali et al., 2019). In recent years, precision medicine has been gradually applied to the cardiovascular field along with the development of gene medicine. Currently, the following diseases are considered: common cardiovascular diseases, such as hypertension (Sun et al., 2017a; Padmanabhan and Joe, 2017; Savoia et al., 2017); diagnosis and treatment of uncertain diseases, such as angina and coronary artery disease (Ladapo et al., 2017; Niccoli et al., 2017); high-mortality diseases and/or interventions are complex and expensive, such as dilated cardiomyopathy and cardiac resynchronization therapy (Halliday et al., 2017). It is worth emphasizing that these methods involve new molecular and genetic diagnostic methods (Padmanabhan and Joe, 2017; Savoia et al., 2017).

Maternally expressed gene 3 (MEG3) is an imprinted gene with maternal expression that encodes a noncoding RNA with a length of −1,600 nt (Tian et al., 2015) located at chromosome 14q32 (Zhang et al., 2010). The involvement in human disease processes is increasingly reported. For example, MEG3 functions as a competing endogenous RNA to regulate cancer progression, reduces mitochondrial-derived apoptosis (Wang et al., 2021a), participates in the regulation of tumor drug resistance (Yu et al., 2020), participates in glaucoma onset (Sun et al., 2018), etc., This noncoding RNA is not only involved in the pathogenesis of many diseases but also found in an increasing number of mechanisms. For instance, it participates in cell migration and proliferation, promotes cell apoptosis, inhibits cell autophagy activity, and inhibits inflammatory factors (Li et al., 2017; Han et al., 2020; Yang et al., 2022). Cardiovascular disease researchers are increasingly interested in MEG3. Hongchun Wu’s group reported that MEG3 exists in mouse myocardial cells and myocardial fibroblasts and mainly plays a role in the myocardial nucleus (Wu et al., 2018). Thum’s team reported that MEG3 plays a key role in promoting cardiac fibroblast fibrosis (Piccoli et al., 2017). The inhibition of Meg3 prevents cardiac fibrosis and diastolic dysfunction (Piccoli et al., 2017), and the downregulation of MEG3 protects myocardial cells against I/R-induced apoptosis through the miR-7-5p/PARP1 pathway (Zou et al., 2019) Chao He et al. found in their study that MEG3, as a ceRNA, inhibits miR-9, affects the phenotype of MEG3-mediated vascular endothelial cells (He et al., 2017).

Currently, studies on lncRNA MEG3 in cardiovascular diseases are relatively rare, and there is still a large space for exploration. This paper will review the research progress of MEG3 in the functional mechanism, other systemic diseases and cardiovascular diseases and find its great potential in the prevention and treatment of cardiovascular diseases.

## 2 Functional mechanism

This paper focuses on the role of lncRNA MEG3 in cardiovascular disease. Although so far, there have been few studies on the effects of lncMEG3 on cardiovascular diseases, most of which focus on tumor diseases, from the perspective of functional mechanism, the regulatory mechanisms involved in MEG3 are also common in cardiovascular diseases, suggesting that MEG3 may be involved in the occurrence and development of cardiovascular diseases through these pathways. Therefore, the second part of this paper reviews MEG3 from the functional mechanism.

### 2.1 Signaling pathway

#### 2.1.1 Regulation of the P53 signaling pathway

**FIGURE 1:**
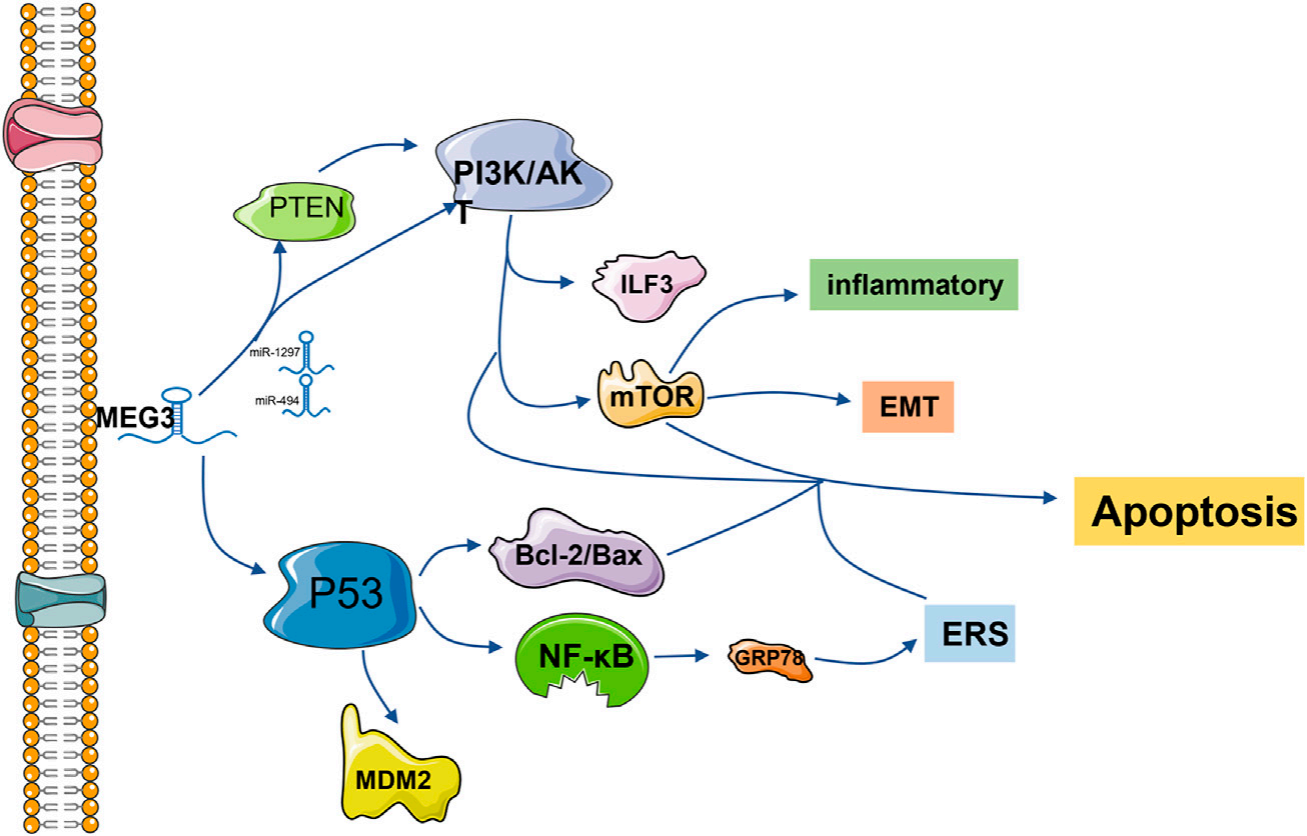
lncRNA MEG3 is frequently involved in endoplasmic reticulum stress, apoptosis, inflammation and epithelial-mesenchymal transformation *via* the P53 and PI3K/AKT pathways. The common downstream pathways are MDM2, NF-κB, Bcl-2/Bax, mTOR and other protein pathways. Regulation is also accomplished by competitively binding downstream protein pathways with miRNA.

The p53 protein is a transcription factor that activates cell cycle arrest, DNA repair and apoptosis in response to stress and DNA damage (Kruiswijk et al., 2015). The 27 known splicing variants of MEG3 contain variable intermediate exons with common exons entraining at the 50 (E1-E3) and 30 (E10-E12) ends, and their ability to stimulate the p53 pathway varies (Yu et al., 2020). Based on this, MEG3 can regulate cell physiology by regulating p53 and downstream signaling pathways and play a role in a variety of diseases. In vascular endothelial cells, the p53 signaling pathway is the most significantly regulated pathway after MEG3 knockout. Downregulation of MEG3 leads to phosphorylation of p53 at serine 15 and weakens its binding with multiple p53 target gene promoters, such as MDM2, resulting in accumulation of p53. Meanwhile, MEG3 loss leads to high expression of P21, induces p53 cell cycle arrest, reduces cell proliferation, promotes cell apoptosis, and aggravates cardiac dysfunction after pressure overload in mice. Early studies have shown that the loss of p53 in the vascular endothelium can reduce apoptosis of endothelial cells and protect cardiac dysfunction after pressure overload in mice (Deng et al., 1995; Gartel and Radhakrishnan, 2005; Gogiraju et al., 2015; Shihabudeen Haider Ali et al., 2019). In tumor cells, lncRNA MEG3 inhibits the proliferation and metastasis of breast, gastric, liver and pancreatic cancer cells by activating the p53 signaling pathway (Chen et al., 2016; Hu et al., 2016; Wei and Wang, 2017; Huang et al., 2022). Especially in hepatocellular carcinoma cells, MEG3 activates the NF-κB signaling pathway and then activates the p53 pathway and upregulates the expression of the ER stress-related protein GRP78, resulting in ER stress (Chen et al., 2016). Xueling Li’s group found that lncMEG3 was downregulated around the infarcted myocardial tissue, which directly acted on p53 and reduced its expression level. Meanwhile, the expression of ERS-related protein GRP78 decreased, which proved that lncRNA MEG3 in myocardial cells was awaiting ERS-mediated apoptosis through the p53 pathway (Li et al., 2019). On the other hand, inhibition of endogenous Meg3 in mouse fibroblasts reduced the expression of Mmp-2 at promoters by inhibiting p53 binding but had no effect on cell apoptosis or proliferation (Piccoli et al., 2017). Western blotting was used to detect the proteins in the pathway, and it was found that p53 was regulated by MeG3, which then regulated the apoptosis of adipose stem cells (adScs) through the Bcl-2/Bax pathway (Shi, 2020). In mouse neurons, overexpression of Meg3 induces p53 and enhances its transcriptional activity, leading to increased cell death (Yan et al., 2016) (Figure 1).

#### 2.1.2 Regulation of the PI3K/Akt signaling pathway

The phosphatidylinositol 3-kinase (PI3K)/protein kinase B (PKB/AKT) signaling pathway is one of the core signaling pathways involved in regulating cell growth, proliferation, apoptosis and other processes (Yang et al., 2019a). Studies have shown that inhibition of the PI3K/AKT signaling pathway can inhibit cell proliferation and promote cell apoptosis (Okano et al., 2000; Oyama et al., 2007; Feng et al., 2016). LncRNA MEG3 has a negative regulatory effect on the PI3K/Akt signaling pathway. LncRNA MEG3 expression was significantly increased in the temporal cortex of rats with subarachnoid hemorrhage and in the cerebrospinal fluid of patients with subarachnoid hemorrhage, while the expression of the PI3K/Akt signaling pathway was decreased, which promoted neuronal apoptosis (Liang et al., 2018). At the same time, the upregulation of MEG3 can improve cognitive dysfunction by inhibiting the PI3K/Akt signaling pathway and then inhibiting the activation of hippocampal astrocytes (Yi et al., 2019). MEG3 overexpression of the inactivated PI3K/Akt signaling pathway can inhibit the growth of cervical cancer HeLa cells and breast cancer cells, thus achieving anticancer effects (Wang et al., 2017; Zhu et al., 2019).

PI3K/AKT can play a regulatory role alone and crosstalk with the mTOR signaling pathway to regulate various pathological processes (Rosenbloom et al., 2013). It is through this signaling axis that MEG3 inhibits the TNF-α-induced inflammatory response (Tang et al., 2021). In endometrial cancer cells, MEG3 regulates the PI3K/m-TOR signaling pathway to regulate cell cycle progression, thus regulating apoptosis (Sun et al., 2017b). MEG3 enhances adenosine-induced hepatocellular carcinoma cytotoxicity by downregulating ILF3 and activating this signaling axis (Pu et al., 2019). MEG3 also plays a positive role as a potential target for the treatment of epilepsy by activating this pathway to reduce proinflammatory factors, oxidative stress and apoptosis of hippocampal neurons in rats (Zhang et al., 2020). In rat and cell models of diabetic retinopathy, overexpression of MEG3 inhibits endothelium-stromal transformation by inhibiting the PI3K/Akt/mTOR signaling pathway (He et al., 2021).

The tumor suppressor PTEN, a central negative regulator of the PI3K/Akt signaling pathway, is often mutated in cancer and loses its tumor suppressor function (Leslie, 2012; Wang et al., 2018). It has been reported that both MEG3 and PTEN are downregulated in ovarian cancer cells, which promotes tumor cell proliferation and inhibits cell apoptosis. LncRNA MEG3 regulates PTEN/PI3K/AKT to play an anti-proliferation role in hemangioma cells and testicular germ cell tumor cells through sponge adsorption of miR-494 and miR-1297, respectively.

Experiments have proven that upregulation of MEG3 can sponge miR-27a-3p, upregulate IGF1 and activate the PI3K/Akt signaling pathway, thus promoting osteogenic differentiation (Liu et al., 2019a) (Figure 1).

### 2.2 MEG3 induces apoptosis

Apoptosis is a programmed cell death that occurs after many stimuli, infections or injuries and plays a key role in normal physiological processes such as embryogenesis and adult tissue homeostasis (Morana et al., 2022). With the advancement of research on MEG3, it has been found that it has an obvious regulatory effect on the molecular mechanism of cell apoptosis, thus participating in a variety of diseases.

MEG3 down-regulation can regulate FOXO1 and FOXO4 to promote apoptosis through competitive binding of miR-361-5P and miR-23b-3p respectively (Tsao et al., 2022), (Wang et al., 2019a), (Wang et al., 2021b). H_2_O_2_ induces oxidative stress in adipose stem cells (adScs), thus increasing the apoptosis rate of adScs. MeG3 silencing can reduce H_2_O_2_-induced apoptosis, while MeG3 overexpression can aggravate apoptosis (Shi, 2020). MEG3 recovery can reduce β-catenin and cyclin D1 in melanoma cells, improve GSK-3 β levels *in vitro*, block Wnt signaling activity, inhibit cell proliferation, migration and invasion, and trigger cell apoptosis (Li et al., 2018a).

MEG3 functions as a ceRNA to regulate apoptosis by sponging different miRNAs (Moradi et al., 2019). Activation of Sema3A, a member of the Sema family, may disregulate a series of apoptosis-related regulatory factors, thus promoting apoptosis (Vadasz and Toubi, 2014; Cheng et al., 2019). MEG3 inhibition significantly increased the expression of miR-424-5p, decreased the expression of Sema3A, increased cell viability, and reduced cell apoptosis (Xiang et al., 2020). As an endogenous sponge, MEG3 inhibits miR-223 function, increases NLRP3 expression and promotes endothelial cell apoptosis through sequence complementation (Zhang et al., 2018). Overexpression of MEG3 inhibits miR-141-3p, activates RNA binding motif single stranded protein 3 (RBMS3), and promotes apoptosis of breast cancer cells (Dong et al., 2021a). Overexpression of MEG3 and miR-376B-3p inhibits the tumogenesis of PDFS cells and promotes cell apoptosis (Zhu et al., 2020). In the *In vitro* AP model, MEG3 promotes cell apoptosis through the MEG3/miR-195-5p/FGFR2 signaling axis (Chen and Song, 2021). MEG3 has been reported to regulate miR-21, target Caspase-8 to regulate proliferation and apoptosis of psoriatic epidermal cells, and inactivate the PI3K/Akt pathway to promote apoptosis in breast cancer cells (Jia et al., 2019; Zhu et al., 2019). Mir-361–5p. In the research of Shen B’s group, it is suggested that MEG3 regulates miR-361–5p and promotes cell apoptosis (Shen et al., 2019). In laryngeal cancer cells, overexpressed MEG3 specifically binds and negatively regulates miR-23a, activates apoptotic protease activator factor-1 (APAF-1), and thus activates Caspase-9 and Caspase-3, leading to apoptosis (Zhang et al., 2019b). MEG3 overexpression leads to reduced apoptosis of intestinal ganglion cells and increased cell viability, and the upregulation of the MEG3 downstream gene miR-211-5p will counteract this effect. Further studies found that the increased expression of GDNF reversed the upregulation effect of miR-211-5p. In summary, hypoxia induced intestinal ganglion cell apoptosis through the MEG3/miR-211-5p/GDNF axis (Xia et al., 2019). MEG3 was found to be highly expressed in the blood and placental villus tissues of gestational diabetes mellitus (GDM). MEG3 knockdown significantly increased the viability of human chorionic trophoblast HTR-8/SVneo cells and reduced cell apoptosis. Inhibition of miR-345-3p negates all observed physiological effects of MEG3 downregulation on HTR-8/SVneo cells (Zhang, 2019). In HCC cells, MEG3 overexpression can sponge out miR-9-5p and upregulate the expression of SOX11, and then observe apoptosis-related changes of Bcl-2 and caspase-3, suggesting that MEG3 can promote the apoptosis of HCC cells (Liu et al., 2019b). For thyroid cancer cells resistant to 131I, low MEG3 expression is not good news. Downregulated MEG3 can promote cell apoptosis by upregulating miR-182 expression and reducing the therapeutic effect of 131I in TC cells (Liu et al., 2018). miR-205-5p has been identified as the downstream gene of MEG3 and is negatively regulated by MEG3, promoting cell apoptosis (Tao et al., 2020). MEG3 inhibition negatively regulates miR-145-5p, resulting in a decreased apoptosis ability of macrophages (Sun et al., 2020). In summary, MEG3, as a ceRNA, mostly has a negative regulatory effect on miRNA and then positively regulates cell apoptosis through the downstream signaling pathway of miRNA.

**FIGURE 2:**
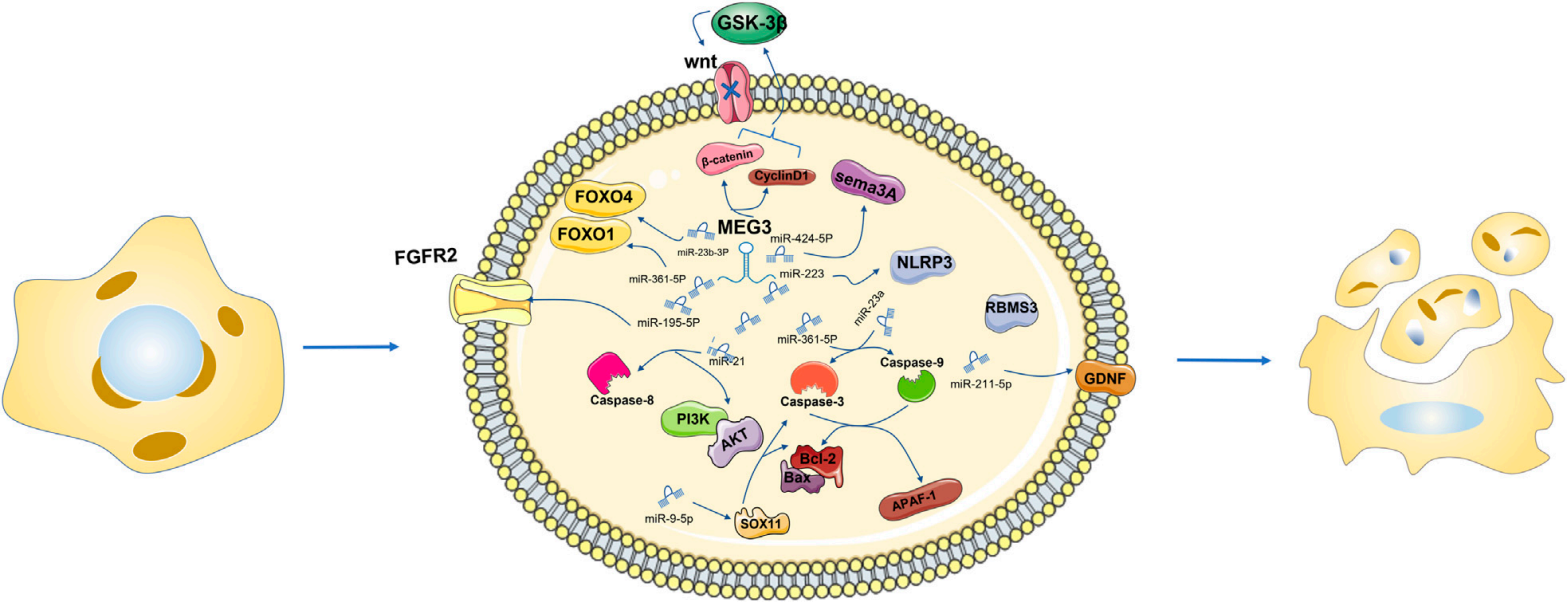
Downregulation of MEG3 can directly or through miR-361-5p mediate the downregulation of FOXO1, promote cell apoptosis, negatively regulate the expression level of miR-23b 3p, and negatively regulate the expression level of FOXO4 in the same family to promote cell apoptosis. MEG3 recovery can reduce β-catenin and CyclinD1, improve the level of GSK-3β *in vitro* culture, block the activity of the Wnt signaling pathway, and then inhibit cell proliferation, migration and invasion, triggering cell apoptosis.

MEG3 inhibition significantly increases the expression of miR-424-5p, reduces the expression of Sema3A, increases cell viability, reduces cell apoptosis, inhibits the function of miR-223, and increases the expression of NLRP3. Overexpression of MEG3 can inhibit miR-141-3p, activate RBMS3, and promote cell apoptosis. MEG3 promotes cell apoptosis through the MEG3/miR-195-5p/FGFR2 signaling axis. MEG3 can regulate miR-21, target Caspase-8 to regulate cell proliferation and apoptosis, and inactivate the PI3K/Akt pathway to promote cell apoptosis. Bcl‐2 overexpression was detected in the siRNA‐MEG3 and miR-361–5P inhibitor groups. MEG3 regulates miR-361–5p and promotes apoptosis. MEG3 overexpression and negative regulation of miR-23a activate APAF-1, thereby activating caspase-9 and caspase-3, leading to cell apoptosis. MEG3 gene knockout significantly increased the viability of HTR-8/SVneo cells and reduced cell apoptosis. MEG3 overexpression can sponge miR-9-5p and upregulate the expression of SOX11, and changes related to apoptosis of Bcl-2 and Caspase-3 were observed. Downregulation of MEG3 can promote cell apoptosis by upregulating the expression of miR-182 (Figure 2).

### 2.3 Cell autophagy

Autophagy refers to the transport of intracellular components to lysosomal chambers for degradation and circulation. To date, autophagy has been defined as three types: macrophage, microphage and chaperone-mediated autophagy, which can be “body autophagy” with nonselective degradation of autophagosome content and “selective autophagy” with elimination of single cell components (Saftig et al., 2008; He and Klionsky, 2009; Galluzzi et al., 2017; Dikic and Elazar, 2018; Gohel et al., 2020). Autophagy is an important function for maintaining cell and body homeostasis, and the basal level of autophagy is important for maintaining normal cellular homeostasis. Therefore, strict regulation of autophagy to induce it when needed plays a crucial role in health and disease (Botti et al., 2006; Meijer and Codogno, 2006; Yorimitsu and Klionsky, 2007; Cheng, 2019). The regulation of LncRNA MEG3 on autophagy is mainly positive regulation, and this effect has been verified in a variety of diseases and multiple pathways. FOXO1 belongs to the forkhead box protein family, which can regulate a variety of biological characteristics including apoptosis, autophagy, proliferation (Kitamura et al., 2005; Kobayashi et al., 2012; Zhang et al., 2016; Li et al., 2021). Downregulation of MEG3 or FOXO1 can lead to decreased autophagy of INS-1 cells induced by high glucose.^1^During pulmonary fibrosis, downregulation of MEG3 activates the Hedgehog (Hh) signaling pathway and inhibits autophagy activity of A549 cells (Gao et al., 2022). Autophagy exists in tumor cells, and downregulation of MEG3 can partially inhibit autophagy in lung cancer cells and can also regulate ATG3 activity to inhibit autophagy in epithelial ovarian cancer cells. (Xia et al., 2018), (Xiu et al., 2017). MiR-7-5p was found to play an active role in autophagy in cardiomyocytes of patients with ventricular septal defects, the binding sequence of MEG3 was found in the 3′UTR of miR-7-5p, and the predicted binding site of miR-7-5p was also found in the 3′UTR of EGFR. Upregulated MEG3 reverses myocyte autophagy through the miR-7-5p/EGFR axis (Kitamura et al., 2005; Kobayashi et al., 2012; Zhang et al., 2016; Li et al., 2021). Low expression of lncRNA MEG3 upregulates miR-543 to regulate autophagy in the IDO signaling pathway (Wang et al., 2021c). continued downregulation of MEG3 leads to IFN-γ induction of autophagy in infected macrophages (Pawar et al., 2016). LncRNA MEG3 enhances TNF-α-induced autophagy by inhibiting the PI3K/AKT/mTOR signaling pathway while inhibiting autophagy through the Beclin-1 signaling pathway (Pu et al., 2019; Tang et al., 2021).

### 2.4 Inflammation

Inflammation is a double-edged sword with many functions that can be seen everywhere in the process of injury and the repair of disease. MEG3 regulates inflammation in a number of ways. KLF4, an important regulator of macrophage polarization, was decreased in M1 macrophages and significantly increased in M2 macrophages, and the expression of macrophage proinflammatory genes with KLF4 knockout was increased (Liao et al., 2011). Inhibition of MEG3 can regulate the secretion of microglia inflammatory cytokines by inhibiting M1 polarization and promoting M2 polarization through KLF4 (Li et al., 2020a). M2 macrophages themselves have certain anti-inflammatory activity. MEG3 can regulate the miR-20b-5p/CREB1 axis and induce M2 macrophage-derived extracellular vesicles (M2-EVs) to reduce the inflammatory response (Wang et al., 2021d). Upregulation of MEG3 in ulcerative colitis (UC) can enhance the protective effect of M2-macrophage-derived extracellular vesicles (M2-evs) against UC and reduce inflammation (Wang et al., 2021d). MEG3 upregulation in methylene blue (MB)-induced peripheral nerve axons alleviates pain and inflammation associated with obstructive sleep apnea by inhibiting P2X3 protein expression (Li et al., 2018b).

Inflammasomes are multimeric protein complexes that are assembled in the cytosol upon sensing pathogen-associated molecular patterns (PAMPs) and danger-associated molecular patterns (DAMPs) (Strowig et al., 2012). AIM2 is one of the key proteins that regulates the formation of the inflammasome. Overexpression of MEG3 regulates the action of AIM2 on caspase1 signaling to trigger an inflammatory response (Liang et al., 2020). After sensing multiple molecular patterns, the n-terminal pyrin fragment (PYD) of NLRP3 serves as a scaffold to nucleate apoptosis-associated spotted proteins and recruit caspase preproteins to the inflammasome (Cai et al., 2014; Lu et al., 2014). MEG3 increases NLRP3-induced inflammation by inhibiting miR-223 or miR-7A-5p (Zhang et al., 2018; Meng et al., 2021). Upregulated MEG3 increases the CXCL12/CXCR4/Rac1 axis and TLR4/NF-κB pathway by inhibiting miR130A-5P, aggravating neuroinflammation (Dong et al., 2021b). Under the condition that IL-1β induces chondrocytes to simulate OA, MEG3 regulates the miR-93/TGFBR2 axis to regulate the inflammatory response and delay the OA process (Chen et al., 2021). miR-181b is an important gene in many human diseases. Experiments verified that lncRNA MEG3 directly regulates the level of miR-181b by binding the 3′UTR of miR-181B and reduces the levels of TNF-α and IL-1B in the serum and cerebrospinal fluid of ICH rats (Xie et al., 2021). MEG3 upregulation inhibits the expression of miR-146a and LET-7I, and the inflammatory factors IL-1β, IL-6 and TNF-α decrease synchronously, alleviating the inflammatory response in ankylosing spondylitis (Li et al., 2020b), (Ma et al., 2020). Acute pancreatitis (AP) is marked by severe inflammation. In the *In vitro* model of AP, the expression levels of MEG3 and FGFR2 are decreased, while the expression levels of miR-195-5p are increased. MEG3 promotes inflammatory responses through the MEG3/miR-195-5p/FGFR2 signaling axis (Chen and Song, 2021). MEG3 overexpression can downregulate miR-34a, enhance SIRT1 protein expression, inhibit the secretion of the inflammatory factors IL-1β, IL-6 and TNF-α, and inhibit liver inflammation by targeting SIRT6 with EZH2 (Tong et al., 2019; Zou et al., 2022).

### 2.5 Endoplasmic reticulum

During a large amount of genetic and environmental damage, the er of the cell is hindered in its ability to properly fold and translate postmodified proteins, leading to misfolding of proteins in the organelles, known as “er stress”. When a large number of misfolded proteins are present, an intracellular signaling pathway called the “unfolded protein response” (UPR) performs transcription and translation functions, restoring homeostasis. Endoplasmic reticulum stress and UPR defects are increasingly becoming key factors in human diseases, and the regulatory role of MEG3 in this aspect has begun to emerge (Sevier and Kaiser, 2002; Tu and Weissman, 2004; Ron and Walter, 2007; Wang and Kaufman, 2012). After MEG3 is downregulated in rectal cancer cells and MEG3 is restored, UPR response-related proteins, including GRP78, ATF6 and CHOP, are highly induced, and the cell apoptosis rate increases. The above reactions can be alleviated by simulated administration of miR-103A-3p. It can be concluded that MEG3 induces ER stress to promote the apoptosis of rectal cancer cells, and the interaction between miR-103A-3p and MEG3 negatively regulates this process (Wang et al., 2021e). At the same time, MEG3 upregulation activates NF-κB signaling to initiate ERS (Bao et al., 2020). As also mentioned above, MEG3 is involved in ER stress in HCC cells and cardiomyocytes by activating the p53 signaling pathway.

### 2.6 Cell proliferation, migration and invasion

Genomic deletion of QKI-5, a member of the RNA (STAR) signal transduction activator protein family, promotes proliferation and dedifferentiation of cancer cells (Fu and Feng, 2015; He et al., 2016). Overexpression of MEG3 inhibits the expression of miR-9-5p and then upregulates QKI-5 to inhibit the proliferation, migration and invasion of prostate cancer cells (Wu et al., 2019). MEG3 overexpression inhibits the proliferation, invasion and migration of pituitary tumor cells, which is achieved by negatively regulating miR-23b -3p and miR-23b -3p negatively regulating FOXO4 (Wang et al., 2021b). MEG3, miR-376B-3p and HMGA2 form a signaling axis to regulate the invasion of pituitary tumor cells (Zhu et al., 2020). MEG3 and miR-361-5p regulate FoxM1 *in vitro* and inhibit the proliferation, migration and invasion of osteosarcoma cells (Shen et al., 2019). In a study of gastric cancer, MEG3 overexpression inhibits the expression of miR-21 and inhibits cell proliferation and metastasis (Dan et al., 2018).

For thyroid cancer cells resistant to 131I, low expression of MEG3 means increased expression of miR-182 and promotion of cell proliferation, which is detrimental to the treatment effect of 131I for thyroid cancer (Liu et al., 2018).

### 2.7 EMT

EMT was originally defined as the developmental program of epithelial cells during gastrula formation or movement of various cell types from the neural crest to distant sites in the embryo (Nieto et al., 2016; Dongre and Weinberg, 2019). In adult epithelial tissue, EMT is involved in the physiological process of wound repair and can be activated under certain pathological conditions, such as fibrosis and cancer (Nieto et al., 2016). In principle, cell phenotypic transitions engineered by EMT are reversible, so that individual cells previously activated by the EMT program can and often do revert to an epithelial state through mesenchymal to epithelial transition (MET) (Lu and Kang, 2019). EMT programs are initiated in cells by paracrine signaling factors to which they are exposed, most notably transforming growth factor-β (TGFβ). In addition, there are various other signals, such as WNT proteins, cytokines, growth factors, and extracellular matrix (ECM)-integrin interactions (Lambert and Weinberg, 2021). In reports on U251 cells, MEG3 overexpression resulted in decreased EMT, decreased expression of n-cadherin, vimentin, snail-1 and β-catenin, and increased expression of e-cadherin in U87 and U251 cells (Gong and Huang, 2020). MEG3 silencing also promotes EMT by regulating the miR-377/PTEN axis (Wang et al., 2019b). Yang et al. reported that the expression of MEG3 induced more mesenchymal cell morphology and increased zeb1/2 expression. Notably, autophagy inhibition can inhibit MEG3-induced EMT (Gao et al., 2018).

## 3 lncRNA MEG3 plays a role in cardiovascular disease

### 3.1 Cardiac fibrosis

**FIGURE 3:**
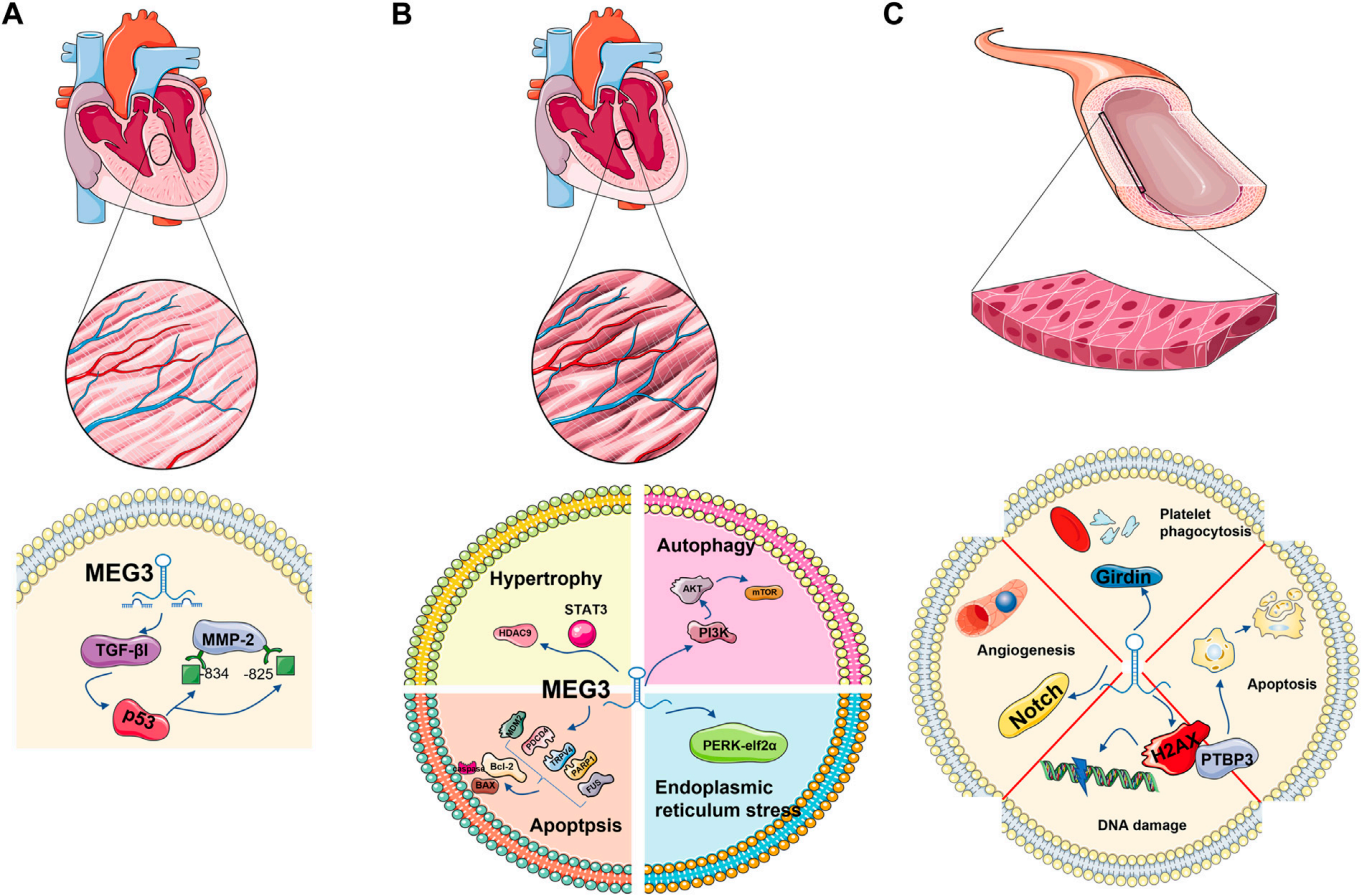
MEG3 plays a role in cardiovascular-related cell types: cardiomyocytes, cardiac fibroblasts and endothelial cells. Regulation of TGE-β1 and P53 in cardiac fibroblasts; It is involved in apoptosis and autophagy in cardiomyocytes. It is associated with autophagy and DNA damage in endothelial cells.

Cardiac fibrosis is a pathologic process involved in almost all cardiac diseases. Cardiac fibroblasts (CFs) are the main coordinators of extracellular matrix (ECM) remodelling after left ventricular pressure overload and play a key role in maintaining cardiac ECM integrity and homeostasis. Upon injury, CFs are activated into myofibroblasts that produce matrix metalloproteinases (MMPs) (Eghbali et al., 1989; Spinale, 2007; Travers et al., 2016; Frangogiannis, 2021). As a member of the MMP family, the increase in MMP-2 leads to the aggravation of fibrosis. LncRNA MEG3 was significantly enriched in myocardial fibroblasts. MEG3 silencing inhibits transforming growth factor (TGF-βI), interferes with the binding of p53 to sites −843 and −825 on the MMP-2 promoter, inhibits the activity of MMP-2, and subsequently decreases the expression of the fibrosis marker Ctgf. Thus, lncRNA MEG3 is coupled with p53 and plays a role in myocardial fibrosis (Piccoli et al., 2017) (Figure 3A).

### 3.2 Myocardial cells

#### 3.2.1 Cardiomyocyte hypertrophy

In the process of cardiac hypertrophy, the role of lncRNA MEG3 cannot be ignored. Jingchang Zhang et al. found that the expression of MEG3 was abundant in cardiomyocytes treated with 150 nM ANG-II, and SH-MEG3 reversed the increase in the surface area of cardiomyocytes induced by ANG-II. Meanwhile, the expression of atrial natriuretic factor (ANF), brain natriuretic peptide (BNP) and β-myosin heavy chain (β-MHC) were decreased. This result preliminarily indicates that MEG3 positively regulates cardiac hypertrophy. After further research, Jingchang Zhang et al. found the promoter of MEG3-STAT3 with a binding relationship at site 2. Activated by STAT3, MEG3 competitively binds and inhibits miR-361-5p, upregulates downstream HDAC9 protein, and has a negative effect on cardiac hypertrophy (Zhang et al., 2019a).

#### 3.2.2 Cardiac autophagy

Several studies have demonstrated that multiple upstream signals can regulate mTOR activity by regulating the phospholipid inositol three kinase protein (PI3K)/kinase B (AKT) signaling pathway (Nishimoto, 2000; Sun et al., 2017b; Wang et al., 2018). Hou et al. (2014) reported that advanced glycation end products inhibited the PI3K/Akt/mTOR pathway to promote cardiac autophagy. miR-7-5p is an important regulator of autophagy in a variety of cells (Song et al., 2016; Cai et al., 2017; Gu et al., 2017). Yinyin Cao et al. reported that MEG3 directly targets miR-7-5p and regulates downstream mTOR signal activation, thus regulating cardiac autophagy (Cao et al., 2019).

#### 3.2.3 Myocardial cell apoptosis

Hongchun Wu et al. reported that in hypoxic cardiomyocytes, p53 affects the expression of Meg3 by directly binding upstream of the Meg3 gene. Meg3 is upregulated by P53 and promotes cell apoptosis by directly binding to FUS protein. At the same time, after MEG3 inhibition, the expression of the pro-apoptotic gene Caspase-3 was decreased, and the expression of the anti-apoptotic gene Bcl-2 was increased, thus inhibiting myocardial cell apoptosis.^23^Ya Zhao et al. demonstrated that MEG3 regulates the expression of Bcl-2 and Caspase-3 proteins through miR-325 3p mediation, negatively regulating the expression of TRPV4 and thus alleviating myocardial cell injury and apoptosis (Zhou et al., 2021). In the study of Bin Yang et al., Bax expression was downregulated in H9C2 cells after oxygen and glucose deprivation (OGD) induction, caspase-3 and Caspase-9 were cleaved, and apoptosis occurred. In this process, MEG3 expression is increased, MDM2 depletion is increased, and the production of p53 is promoted, while the phosphorylation level of AMPK is reduced (Yang et al., 2019b). In the study of Yiwei Chen et al., lncRNA MEG3 expression was significantly increased in AC16 cardiomyocytes induced by high glucose (HG). Meanwhile, downregulation of MEGers.

Three could restore the effects of decreased expression of Bcl-2/Bax and increased expression of Caspase-3, which had beneficial effects on cell viability and apoptosis. Further studies showed that miR-145 directly acted on MEG3 and targeted PDCD4 protein at the 3′UTR binding site, and inhibition of miR-145 would offset the beneficial effects of downregulated MEG3 on HG-treated AC16 cardiomyocytes (Chen et al., 2019). Through the study of H9C2 cardiomyocytes after I/R injury, it was confirmed that downregulation of MEG3 can directly and negatively regulate miR-7-5p, regulate the downstream PARP1 protein and caspase-3 protein of miR-7-5P, and save cell apoptosis (Zou et al., 2019).

ERS induced by myocardial ischemia can induce myocardial cell apoptosis (Wei et al., 2016). *In vivo* experimental studies have shown that inhibition of lncRNA MEG3 can reduce the upregulation of endoplasmic reticulum stress-related proteins after ischemia, including BIP/GRP78, ATF4, and C‐EBP homologous proteins (CHOP) (Li et al., 2019). Decreased levels of reactive oxygen species (ROS) are downstream events of endoplasmic reticulum stress after ischemia, and lenti‐Si lncRNA MEG3 pretreatment can reduce ROS levels. Superoxide dismutase (SOD), an important antioxidant enzyme, is significantly reduced in ischemic myocardium, and downregulation of the MEG3 gene can negatively regulate this effect (Li et al., 2019). Previous studies have shown that the PERK‐eIF2α and Caspase 12 pathways play a dominant role in stress-mediated apoptosis (Groenendyk et al., 2013), and downregulation of lncRNA MEG3 can inhibit ER stress through this pathway. In conclusion, downregulation of MEG3 can inhibit endoplasmic reticulum stress in cardiomyocytes and protect cardiomyocytes (Li et al., 2019).

According to L.-Y. Zhao et al., lncRNA MEG3 can also promote the apoptosis of hypoxic cardiomyocytes through the FoxO1 signaling pathway. FoxO1 is activated in the form of phosphorylation, which affects cell stability, leads to cell disorder, and promotes apoptosis of cardiomyocytes (Liu et al., 2017a; Kinyua et al., 2018). However, MEG3 upregulation in hypoxic cardiomyocytes not only upregulates FoxO1 but also increases the expression of FoxO3a in the same family. The combination of the two can enhance autophagy of cardiomyocytes and aggravate apoptosis of cardiomyocytes (Zhao et al., 2019).

#### 3.2.4 Survival and proliferation of cardiomyocytes

Jinwen Su et al. found that the survival and proliferation of cardiac progenitor cells (CPCs) were inhibited under hypoxia, and the level of MEG3 significantly increased. Moreover, the cell viability and proliferation potential of cardiomyocytes increased after MEG3 inhibition. With the inhibition of MEG3, the expression level of miR-22 is increased, the expression level of HMGB1 protein, the next level target of MEG3, is decreased, and cell viability and proliferation are restored. In summary, MEG3/miR-22/HMGB1 has been confirmed to play a role in the survival and proliferation of cardiomyocytes after hypoxia (Su et al., 2018) (Figure 3B).

### 3.3 lncRNA MEG and vasculature

#### 3.3.1 Angiogenesis

Liu et al. showed that MEG3 silencing can negatively regulate the Notch pathway to promote endothelial cell proliferation and angiogenesis (Liu et al., 2017b). In line with this, Chao He et al. found in their study that MEG3, as a ceRNA, inhibits miR-9, affects the phenotype of MEG3-mediated vascular endothelial cells (VECs), and inhibits cell proliferation and *in vitro* angiogenesis. However, the restoration of miR-9 can only rescue part of the angiogenesis inhibition caused by MEG3, which means that other microRNAs are also involved in this process (He et al., 2017). The experiment of Liu, H. Z et al. reported that the interaction between MEG3 and miR-150-5p was also involved in the angiogenesis of endothelial progenitor cells (Liu et al., 2016), which confirmed this point. In a report on anisomycin inhibition of angiogenesis, the spongy action of lncRNA MEG3 was inhibited and angiogenesis was inhibited *via* the miR-421/PDGFRA axis (Ye et al., 2019).

#### 3.3.2 DNA damage of endothelial cells

DNA double-strand breaks (DSBs) are a serious form of DNA damage. Tail length and tail moment of DNA are positively correlated with DSB burden (Olive and Banáth, 2006; Ribas-Maynou et al., 2012). MEG3 is a DNA damage response gene mediated by the p53 pathway. ^5^According to a report, in endothelial cells, after Meg3 was knocked out by 10 nM GapmeRs (chemically modified antisense oligonucleotides), tail length and tail camber were increased by 1.9 times and 2.7 times, respectively. After Meg3 is reduced by 2 nM GapmeRs, tail length and tail camber are increased by 2.4 times and 2.9 times, respectively (Shihabudeen Haider Ali et al., 2019). Phosphorylation of histone H2AX at Ser-19, a marker of DSB damage and repair (Mah et al., 2010; Sharma et al., 2012; Turinetto and Giachino, 2015), also increases. These data suggest that MEG3 protects DNA from damage in endothelial cells. Further studies have found that knocking down PTBP3 induces the expression of the target gene p53, leading to cell apoptosis, while MEG3 can participate in the DSB process by regulating PTBP3’s action on p53 or directly acting on p53, thus protecting endothelial function (Shihabudeen Haider Ali et al., 2019).

#### 3.3.3 Endothelial cell senescence

LncRNA MEG3 also plays a role in endothelial cell senescence. It has been detected that the competitive adsorption of MEG3 in aging endothelial cells decreases, leading to the upregulation of miR-128 and the targeting of Girdin protein, reducing the expression of Girdin protein. In addition, these processes inhibit platelet phagocytosis of endothelial cells (Lan et al., 2019) (Figure 3C).

## 4 Conclusion

lnc1. The diseases involving lncRNA MEG3 mainly include osteosarcoma, laryngeal cancer, prostate cancer, lung cancer, liver cancer, breast cancer, endometrial cancer, oral squamous cell carcinoma, gastric cancer, pancreatic cancer, colorectal cancer, and other malignant tumors. Metabolic diseases such as diabetic retinopathy; rheumatoid arthritis, ankylosing spondylitis, systemic lupus erythematosus and other immune system diseases; heart failure, hypertension, stroke and other cardiovascular and cerebrovascular diseases. A comprehensive understanding of its mechanism is essential for its safety and efficacy in clinical application.

lncRNA MEG3 plays a preventive and/or therapeutic role in cardiovascular diseases by reducing inflammatory injury, apoptosis and endoplasmic reticulum stress. lncRNA MEG3 is closely related to various pathologic effects of cardiomyocytes, fibroblasts and endothelial cells, and has great clinical application potential in the prevention and treatment of AS, MIRI, hypertension and HFLnc. Although some knowledge has been gained about the benefits of lncRNA MEG3 for cardiovascular disease from experimental data, it is worth noting that its specific underlying mechanisms are still relatively unknown. Therefore, available clinical and pharmacological data are insufficient to evaluate its efficacy.

In addition, lncRNA MEG3 has a profound research foundation in tumor diseases, first showing biomarker value in straight-colon cancer (Wang et al., 2019c), gastric cancer (Ghaedi et al., 2019), breast cancer (Ali et al., 2020) and other cancers (Duan et al., 2016; Zhao et al., 2018; Ali et al., 2020). Further research and technology development are needed to improve its bioavailability and overcome the challenges of its clinical application. Second, MEG3 expression is associated with WHO grade of tumor, old age at diagnosis, Karnofsky performance score (KPS), wild-type isocitrate dehydrogenase (IDH), tumor recurrence and overall survival, which is of prognostic value (Gong and Huang, 2017; Zhao et al., 2018; Buccarelli et al., 2020). Third, enhanced MEG3 expression can increase chemotherapy sensitivity to cisplatin, while SI-RNA silencing of MEG3 can induce cisplatin resistance (Ma et al., 2017), which can be used to measure therapeutic response. At the same time, MEG3 has gradually gained attention in cardiovascular disease, a major human public health problem. It can be a worthy object of study. Based on the research basis of lncRNA MEG3 in cancer diseases, we boldly speculate that MEG3 will play a role in the emerging discipline of cancer heart disease (Momtazmanesh and Rezaei, 2021).
